# DMSO Efficiently Down Regulates Pluripotency Genes in Human Embryonic Stem Cells during Definitive Endoderm Derivation and Increases the Proficiency of Hepatic Differentiation

**DOI:** 10.1371/journal.pone.0117689

**Published:** 2015-02-06

**Authors:** Katherine Czysz, Stephen Minger, Nick Thomas

**Affiliations:** GE Healthcare Life Sciences, The Maynard Centre, Cardiff, Wales, United Kingdom; University of Newcastle upon Tyne, UNITED KINGDOM

## Abstract

**Background:**

Definitive endoderm (DE) is one of the three germ layers which during *in vivo* vertebrate development gives rise to a variety of organs including liver, lungs, thyroid and pancreas; consequently efficient *in vitro* initiation of stem cell differentiation to DE cells is a prerequisite for successful cellular specification to subsequent DE-derived cell types [1, 2]. In this study we present a novel approach to rapidly and efficiently down regulate pluripotency genes during initiation of differentiation to DE cells by addition of dimethyl sulfoxide (DMSO) to Activin A-based culture medium and report its effects on the downstream differentiation to hepatocyte-like cells.

**Materials and Methods:**

Human embryonic stem cells (hESC) were differentiated to DE using standard methods in medium supplemented with 100ng/ml of Activin A and compared to cultures where DE specification was additionally enhanced with different concentrations of DMSO. DE cells were subsequently primed to generate hepatic-like cells to investigate whether the addition of DMSO during formation of DE improved subsequent expression of hepatic markers. A combination of flow cytometry, real-time quantitative reverse PCR and immunofluorescence was applied throughout the differentiation process to monitor expression of pluripotency (POUF5/OCT4 & NANOG), definitive endoderm (SOX17, CXCR4 & GATA4) and hepatic (AFP & ALB) genes to generate differentiation stage-specific signatures.

**Results:**

Addition of DMSO to the Activin A-based medium during DE specification resulted in rapid down regulation of the pluripotency genes OCT4 and NANOG, accompanied by an increase expression of the DE genes SOX17, CXCR4 and GATA4. Importantly, the expression level of ALB in DMSO-treated cells was also higher than in cells which were differentiated to the DE stage via standard Activin A treatment.

## Introduction

Pluripotent stem cells (pSC) possess the remarkable ability to provide an origin for virtually all cell types present in a given organism and *in vitro* differentiation of pSC to hepatocyte-like cells can potentially generate limitless numbers of this valuable cell type [3–5]. This potential holds great promise for research and therapeutic applications in drug development and detection of drug-induced toxicity, and for pharmaceutical and regenerative medicine.

However, the process of differentiation to a specific cell type is very often inefficient and lacks robustness in reproducibility of results. In many instances the satisfactory competence of cells to acquire a certain identity (e.g. definitive endoderm) does not lead to cells being able to subsequently commit further to a desired cell type, even when the necessary growth factors/small molecules are added in a stage-specific manner [6, 7]. Definitive endoderm, one of the three germ layers formed at approximately 15 days during human embryogenesis, gives rise to a variety of organs including liver and consequently methods for efficient DE differentiation *in vitro* are of significant interest. Work by D’Amour et al. in 2005 [1] produced a significant development in definitive endoderm derivation *in vitro*; exposure of human embryonic stem cells (hESC) to 100ng/ml of Activin A in the presence of low concentrations of serum in RMPI 1640 medium primed a high number of cells to acquire DE identity. Activin A was used to mimic Nodal signalling which is crucial during DE development *in vivo*. Following five days in culture DE cells were characterised by expression of SOX17, CXCR4, and FOXA2 markers and by concurrent lack of transcription of the primitive and visceral endoderm markers SOX7 and AFP.

While in subsequent studies numerous additional factors have been added to the DE specification medium in attempts to improve differentiation; e.g. NaButyrate and B27 [2, 8]; Albumin fraction V [9]; FGF4 and BMP2 [10]; Wnt3a and HGF [11]; the use of 100ng/ml of Activin A as a key differentiation agent remains ubiquitous. However, while expression of DE and other differentiation markers were shown to increase, expression of the pluripotency transcription factors OCT4 and NANOG has been reported as being difficult to efficiently down regulate [2, 12, 13], suggesting that upon initiation of the differentiation process, the response of hESC to the differentiating factors may be hindered to some extent.

In this study we describe a novel, simple and robust improvement of differentiation of hESC to DE by addition of DMSO to early stage cultures. Applications of this molecule in cell culture are broad [14–20], with DMSO being used to maintain function of hepatocytes cultured *in vitro* [21] and in hESC-derived hepatic progenitor/hepatoblast formation [2]. We have used the addition of DMSO to Activin A-based medium in the initial stages of DE specification to efficiently and concertedly produce rapid down regulation of hESC pluripotency genes and up regulation of definitive endoderm markers and to enhance the expression of hepatic markers during subsequent differentiation.

## Materials and Methods

### Culture of undifferentiated hESC

The H1 hESC line was acquired from the WiCell Research Institute (Madison, WI), propagated on Matrigel-coated vessels and cultured in mTeSR (both from StemCell Tech). The H7 hESC cell line (WiCell Research Institute) was propagated and maintained on Matrigel coated vessels in X-Vivo10 Medium (Lonza), supplemented with 80ng/ml FGF2 and 0.5ng/ml TGFBI (R&D Systems). hESC were cultured in feeder-free, serum-free conditions and cells were passaged at approximately 80% confluence by treatment with 5mg/ml Collagenase IV for 5 min, followed by wash with PBS and reconstitution in an appropriate volume of medium. If an estimation of an accurate cell number of starting material was required prior to the differentiation process, after collagen treatment one representative cell culture vessel was sacrificed and hESC cells disaggregated with 0.25% Trypsin-EDTA (all from Life Technologies). 10% FBS (PAA) in RPMI 1640 medium (Life Technologies) was then used to stop trypsinization and the number of total and viable cells determined using a NucleoCounter YC-100 (Chemometec). The cells were then washed with PBS, scraped in medium and passaged into new Matrigel coated vessels at a cell density of 0.6x10^5^cells/cm^2^. hESC culture media were changed daily.

### hESC Differentiation

**Definitive endoderm formation**. hESC were passaged into culture vessels (T25 flasks and/or 6, 24, 96 well plates) at 0.6x10^5^cells/cm^2^ and cultured for 2 days. To initiate DE differentiation hESC were washed once with PBS and cultured in RMPI 1640 medium (Life Technologies) supplemented with 100ng/ml of Activin A (R&D Systems) with 0.2% FBS added after 24h. Growth factors and modulators used during optimisation of DE specification were 5ng/ml FGF2, 50ng/ml Wnt3a, 1ug/ml sFRP5, and from 0.25 to 2% DMSO (Sigma). In total, cells were cultured for four days with media changed daily.

**Hepatic specification**. hESC-derived DE cells were washed once with PBS and cultured in KO-DMEM medium and 2%KOSR supplemented with 1mM L-glutamine, NEAA (all from Life Technologies), β-Mercaptoethanol (Sigma), 30ng/ml BMP2, 10ng/ml FGF4 and 0.5%DMSO for 5 days. The resulting hepatoblast-like cells were washed with PBS, trypsynized, plated onto new Matrigel-coated vessels at 0.4x10^5^cells/cm^2^ and differentiated in this culture condition for 3 days but with BMP2 replaced with 10ng/ml HGF (PeproTech). Cells were then washed with PBS and cultured for 6 days in HepatoZYME medium (Life Technologies) supplemented with 2% FBS, 1mM L-glutamine, 2ug/ml Insulin (Roche), 2ug/ml Ascorbic Acid (Sigma), 10^-7^M Dexamethasone (Sigma), 10ng/ml HGF and 10ng/ml OSM (R&D Systems) with medium changes every second day. Cells were cultured for a further 10 days in L-15 medium (Phenol Red-free, Life Technologies) supplemented with 2%FBS, 2ug/ml Ascorbic Acid, 10mM HEPES (Life Technologies), 2ug/ml Insulin, 10^-7^M Dexamethasone, and 10ng/ml OSM with daily medium changes.

### HepG2 culture

The hepatocellular carcinoma HepG2 cell line was continuously cultured in RPMI 1640 medium supplemented with 1mM L-Glutamine, NEAA and 10% FBS. Cells were passaged with 0.25% trypsin for 5min followed by addition of culture medium and plating on poly-D-lysine coated vessels at 0.4x10^5^cells/cm^2^.

### Immunofluorescence analysis

For detection of stage-specific markers, cells were grown and differentiated in 96 well plates (μClear, Greiner). Cells were rinsed twice with PBS, fixed in 4% paraformaldehyde (USB) for 15 min at room temperature (RT) and then washed twice with PBS and blocked for 30 min at RT in 1% BSA (Life Technologies) and 0.1 mg/ml human IgG (Sigma) in perm/wash buffer (BD). Cells were subsequently stained for 2 hours at RT or overnight at 4°C with rabbit anti-OCT4 (Cell Signalling), mouse anti-SOX17 (Abcam) or isotype control antibodies diluted in perm/wash buffer. Cells were washed with perm/wash buffer and incubated at 4°C in the dark with goat anti-mouse-FITC and chicken anti-rabbit-Cy5 (Molecular probes) diluted 1:400 in perm/wash buffer. After 1h of incubation, cells were washed with PBS and incubated with Hoechst 33342 (Life Technologies) for 15 min at room temperature. After final washing with PBS 96 well plates were then imaged with IN Cell Analyzer 2000 (GE Healthcare).

### Flow cytometry analysis

Cells in 6 well plates were washed twice with PBS and treated with 0.25% trypsin-EDTA (Life Sciences) for 5 minutes to obtain single cell suspensions. Trypsin was inactivated with medium containing 10% FBS. Cells were counted, centrifuged at 300g for 5 min, washed twice with PBS and subsequently fixed in 2% paraformaldehyde (USB). Following 15 min incubation at room temperature cells were washed in PBS and in perm/wash buffer (BD) and resuspended at 4x10^6^ cells/ml in blocking buffer comprising perm/wash buffer with 0.1mg/ml human IgG (Sigma) and 10% serum from the species of secondary antibody (Life Technologies). Cells were incubated for 30min at 4°C and 50μl aliquots (2x10^5^ cells) transferred to individual 5 ml polystyrene round-bottom FACS assay tubes. For double staining of cells for OCT4 and SOX17, cells were incubated in perm/wash buffer with mouse anti-OCT4 (Cell Signaling) for 1h at room temperature, following by two washes with PBS and incubation for 1 hour at 4°C with goat anti-mouse-FITC (Molecular Probes) and goat anti-SOX17-APC (R&D Systems). Samples were washed twice and resuspended in 0.2% FBS in PBS in a final volume of 300μl/tube. Separate staining for OCT4 and SOX17 was performed analogously. Cells were analysed on a FACSCalibur flow cytometer (Becton Dickinson) and data analysed using CellQuest software.

### Quantitative real-time reverse PCR

Isolation of total cellular RNA was performed using the illustra RNAspin Mini RNA Isolation Kit (GE Healthcare) and RNA concentrations measured on a NanoDrop 1000 spectrophotometer. 1μg RNA was reverse transcribed using a High Capacity cDNA Reverse Transcription Kit (Applied Biosystems) and TaqMan qPCR performed using unlabelled PCR primers and FAM-based probes (Applied Biosystems) in conjunction with TaqMan Universal PCR Master Mix, No AmpErase UNG (Applied Biosystems). qPCR was carried out on a 7900HT PCR System (Applied Biosystems) with cycling conditions of 95˚C for 10min, 45 cycles of 95°C for 10 sec and 60°C for 1min. Analysis of results was performed using the 7900HT PCR System SDS Software. Relative gene expression data were calculated against GAPDH and β-Actin housekeeping genes and mean and standard derivations reported for triplicate determinations for each sample.

## Results

### DMSO rapidly down regulates pluripotency genes in a concentration-dependent manner during definitive endoderm specification

**Fig 1:**
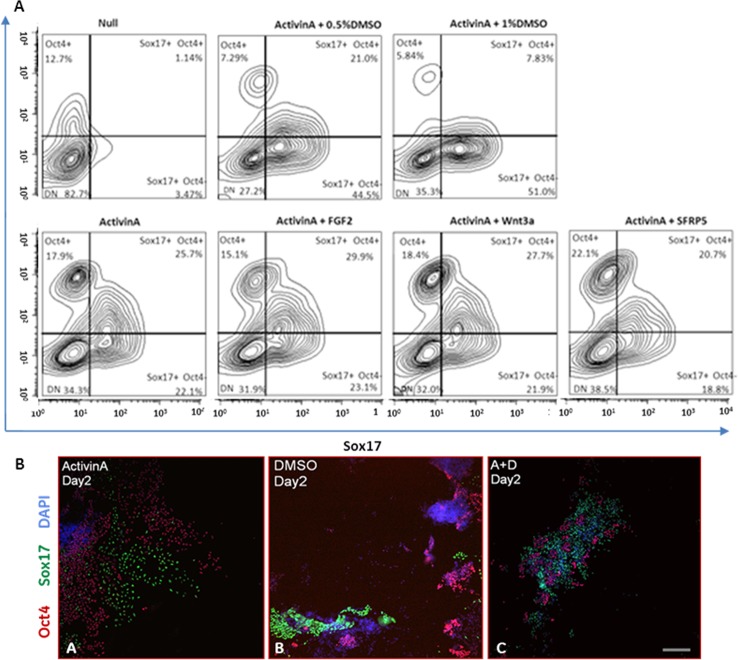
Addition of DMSO to Activin A/low serum-based medium increases the efficiency of definitive endoderm differentiation. (A) Flow cytometry analysis of hESC and DE cells double-stained for the pluripotency marker OCT4 and the endoderm marker SOX17. Pluripotency and differentiation status in all culture conditions tested are shown as contour plots. Only upon addition of DMSO (0.5%, 1%) to Activin A-driven differentiation of hESC was the number of OCT4-positive cells significantly down regulated. Culture with 1% of DMSO yielded the highest number of SOX17+, OCT4- cells. ‘Null’ represents spontaneous differentiation with no differentiating growth factors/reagents added. Each contour plot represents 3 biological replicates pooled together. (B) Immunofluorescence staining of cells at day 2 of DE formation for OCT4 and SOX17 markers with DAPI nuclear staining. Cells cultured in Activin A alone were clearly separated into two widespread populations of SOX17- or OCT4-positive cells (B-A). DMSO in the absence of Activin A does not prime cells to acquire endoderm identity (B-B). Addition of 0.5% of DMSO to Activin A-based, DE-priming medium results in significant decrease of OCT4-positive clusters of cells as early as 48h after DE differentiation was initiated (B-C). Scale bar 100μm.

Nodal signalling is crucial for the specification of definitive endoderm in vertebrates *in vivo* and Activin A is frequently used to recapitulate this signalling pathway *in vitro* to stimulate DE derivation from both embryonic and induced pluripotent stem cells [1]. As this signaling pathway is involved both in maintenance of pluripotency and cellular specification, directed control of staged differentiation very often proves problematic. We routinely evaluated chromosomal integrity and pluripotency of human ES cells (S1 Fig and S2 Fig, respectively). The ability of hESCs to differentiate spontaneously to all of the three germ layers was confirmed by embyoid body formation (S3 Fig). The growth factors FGF2 and Wnt3a have also been reported as being beneficial for DE formation [1, 22, 23] and were included in our optimisation study of Activin A-driven DE formation. In our hands, flow cytometry analysis for the DE marker SOX17 and the pluripotency marker OCT4 (Fig. 1A) showed no significant influence of these growth factors on the efficiency of differentiation as both failed to either enhance Activin A action in priming hESC towards DE differentiation or to decrease numbers of OCT4 positive cells. Similar results were observed with the Wnt3a signalling modulator sFRP5 used here to evaluate Wnt3a influence; while Wnt3a is often included in Activin A-driven DE differentiation, it is the Wnt3a antagonist sFRP5 that is secreted from the foregut part of DE in vivo in order to maintain its identity and avoid differentiation to midgut and hindgut endoderm. In the ‘Null’ culture condition, representing spontaneous differentiation, the majority of cells (82.7%) were OCT4 and SOX17 negative, highlighting the impact of Activin A in directing DE cell specification with 47.8% of cells showing SOX17 expression. However a significant number (43.6%) of Activin A treated cells retained OCT4 expression.

Addition of either 0.5% or 1% of DMSO to the Activin A based medium resulted in significant down regulation of OCT4 and up regulation of the DE marker SOX17 (Fig. 1A) with a resultant doubling of the SOX17^+^/OCT4^-^ population compared to Activin A treatment alone. Although the majority of DE derivations were performed on H1 human ES cell line, differentiations using the H7 human ES cell line as a starting material gave similar observations (data not shown). Interestingly, differentiation of cells to DE analysed at day 2 by immunofluorescence for SOX17 and OCT4 markers showed that DMSO decreased the size of OCT4-positive cell clusters as early as 48h after initiation of DE formation (Fig. 1B). Cells differentiated to DE endoderm which retained OCT4 expression were organized in compacted clusters and only addition of DMSO to Activin A significantly diminished the size of these pluripotent cell clusters (Fig. 1B). In cultures without Activin A, DMSO failed to induce cells to acquire definitive endoderm identity, highlighting the importance of NODAL signalling in this process. Use of DMSO at low concentrations as a solvent for addition of drugs and other compounds to cell culture (≤0.1%) did not have an effect on Activin A-based DE formation (data not shown).

**Fig 2:**
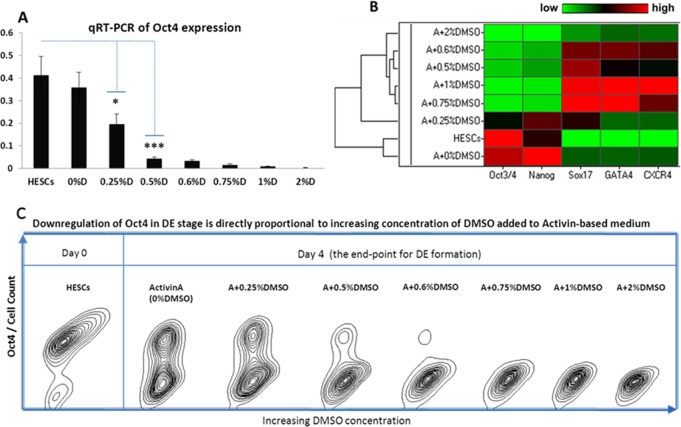
DMSO downregulates pluripotency status in definitive endoderm-primed cells in a concentration-dependent manner. (A) transcriptional and (C) flow cytometry analysis of the pluripotency marker OCT4 in definitive endoderm cells cultured for four days in the presence of 100ng/ml Activin A and varying concentrations of DMSO. (B) Euclidean-based clustering heatmap depicting qRT-PCR analysis of definitive endoderm cells for pluripotency and DE markers (low expression in green, high expression in red). The housekeeping gene GAPDH was used for normalization of the qRT-PCR results. Flow cytometry data represents 3 pooled biological replicates; qRT-PCR data is mean ± 1SD of 3 replicates, Student’s t test: n = 3, (*) p ≤ 0.05, (***) p ≤ 0.001.

**Fig 3:**
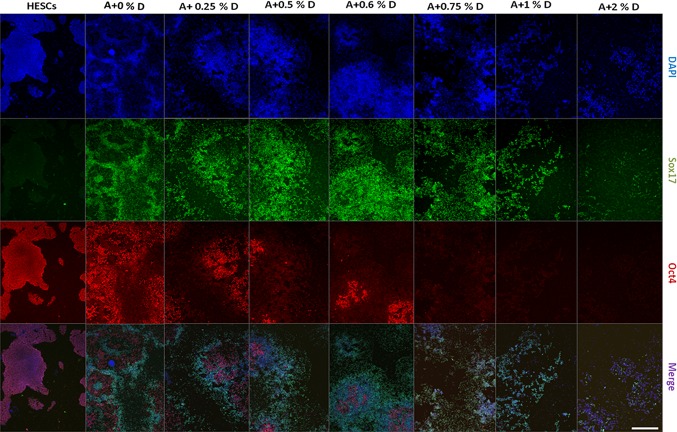
Immunofluorescence staining for SOX17 and OCT4 at day 4 of DE formation. Staining of OCT4 was indirectly proportional to the concentration of DMSO added to Activin A during differentiation of hESC to DE. At ≥ 0.75% DMSO OCT4 staining was abolished while in contrast staining for the DE marker SOX17 was directly proportional to the increasing concentration of DMSO. hESCs; human ES cells. A+0%D; Activin A with no DMSO. A+0.25%D; Activin A with 0.25% DMSO, etc. Nuclei were stained with DAPI. Scale bar, 200μm.

Next, to further investigate the activity of DMSO in enhancing Activin A mediated DE formation, hESC were exposed to varying concentrations of DMSO, ranging from 0.25% to 2% in DE medium. After 4 days of DE specification a strong negative correlation between expression of OCT4 and increasing concentration of DMSO was observed (Fig. 2A & C). Surprisingly, analysis of additional markers (Fig. 2B) revealed that cells cultured in DE medium without DMSO showed high level of NANOG expression when compared to undifferentiated hESC which supports an observation of NANOG being a transcription factor which is often more difficult to downregulate than OCT4. Hierarchical cluster analysis grouped undifferentiated cells and the basic (ActivinA+0%DMSO) DE culture condition into one cluster. The second primary cluster comprised DE culture conditions where differentiating media contained varying concentrations of DMSO. Amongst these the lowest and highest (0.25% and 2%) concentrations were ranked at either side of the optimal concentration group with the latter producing maximal down regulation of OCT4 and NANOG. These data were confirmed by immunofluorescence staining (Fig. 3) which also provided additional insight into optimisation of DMSO concentrations. While high DMSO concentrations produced the desired concurrent high expression of SOX17, CXCR4 and GATA4, cell survival was compromised at concentrations above 0.75%. DMSO concentrations of 0.5% or 0.6% produced significantly increased SOX17 staining and reduced OCT4 staining without impact on cell numbers or morphology.

Although the full role and action of DMSO in cell culture and differentiation remains unknown, it is generally acknowledged that this small molecule may have histone deacetylase inhibitor activity [24] and can convert and/or maintain chromatin in a less compacted state and thus more available for transcription [25]. The findings from this study support a hypothesis in which addition of defined concentrations of DMSO above those commonly used for compound solvation purposes to Activin A-based DE differentiation medium enables enhanced expression of DE-priming genes thereby positively affecting the transcription machinery orchestrating formation of this germ layer and may additionally directly or indirectly contribute to improving the efficiency of downstream differentiation processes.

**Fig 4:**
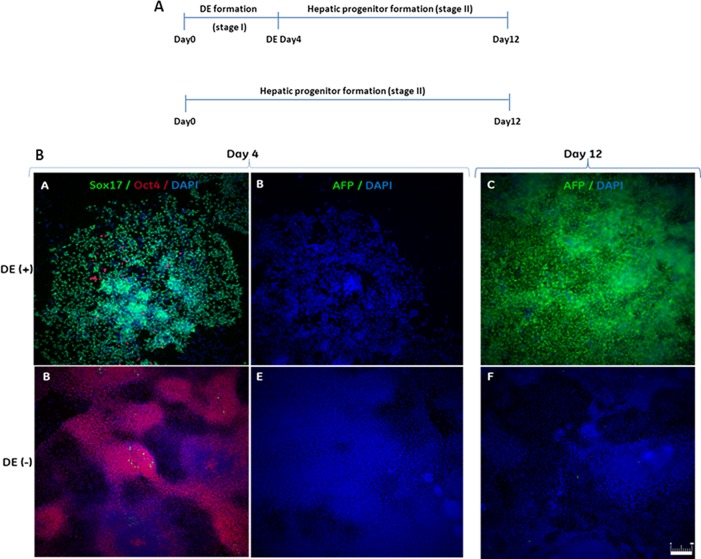
Differentiation through definitive endoderm (DE) is necessary for stage-specific hepatoblast derivation. (A) Schematic overview of differentiation processes. (B) Immunofluorescence analysis of definitive endoderm (day 4) and hepatic progenitors (day 12). hESC that transitioned through the definitive endoderm stage (DE+) were strongly stained for the DE marker SOX17 at day 4 and for the hepatic progenitor marker AFP at day 12. On the contrary, cells cultured directly in hepatic progenitor medium (DE-) failed to acquire DE identity and did not show AFP expression. Scale bar, 200μm.

**Fig 5:**
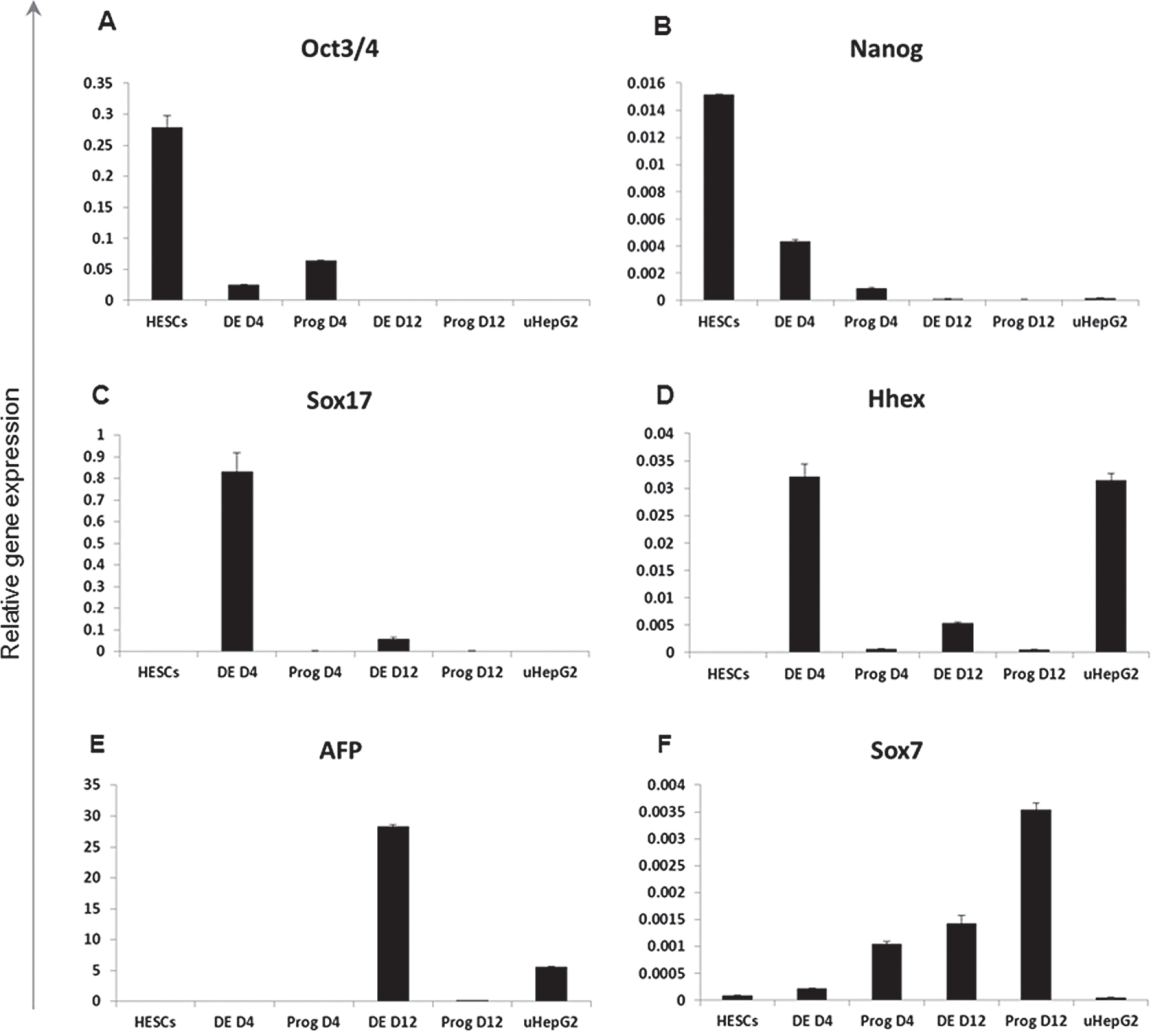
Analysis of transcriptional changes in cells primed to the stage of hepatoblasts with or without prior DE specification. hESCs; human ES cells. DE; definitive endoderm. DE D4; cells transitioned through DE stage and harvested at day 4. Prog D4; cells cultured directly in hepatic progenitor medium and harvested at day4. DE D12: cells transitioned through DE and harvested at day 12. Prog D12; cells cultured directly in hepatic progenitor medium and harvested at day 12. The housekeeping gene GAPDH was used for normalization of the qRT-PCR results. uHepG2; internal control for efficiency of hepatoblast formation. qRT-PCR data are mean ± 1SD of 3 biological replicates.

To confirm that formation of DE via the NODAL signalling pathway is a prerequisite for subsequent hepatic specification, hESC were exposed to either Activin A + 0.5% DMSO DE-inducing signals or to conditions that directly stimulate hepatoblast formation. After four days of culture cells from both conditions were differentiated further to hepatoblasts (Fig. 4A). At each of the indicated time-points cells were harvested and analysed by qRT-PCR and immunocytochemistry. The HepG2 cell line was used as a positive control for hepatoblast marker expression analysis. Real-time quantitative reverse transcription PCR analysis confirmed distinct gene expression profiles between the two culture conditions (Fig. 5). Widespread immunoreactivity for OCT4 was detected in cells that did not transition through the definitive endoderm stage (Fig. 4B; DE-) contrasting with relatively low expression of this transcription marker revealed by qRT-PCR, therefore suggesting its decreased level of expression per single OCT4-positive cell. Importantly, cells which were not exposed to the definitive endoderm culture condition were also negative for expression of the DE-specific genes SOX17 and HHEX at day 4 of specification which contrasted with high levels of expression of these markers in cells exposed to the DE-priming condition (Fig. 5C,D, S4 Fig). Undetectable AFP expression and low SOX7 expression in this culture condition at day 4 suggests that the SOX17- and HHEX-positive cells committed to definitive and not to the visceral endoderm cell lineage [1]. Moreover, fetal hepatic identity was acquired by cells by the end of stage two of differentiation only when pluripotent stem cells were transitioned first through the stage of definitive endoderm, highlighting the necessity of activating the Nodal signalling pathway before pursuing further cellular differentiation of cell types which are derived from this germ layer (Fig. 4B, Fig. 5E).

### Priming cells to the stage of definitive endoderm is crucial for growth factor-based specification to the hepatic lineage

**Fig 6:**
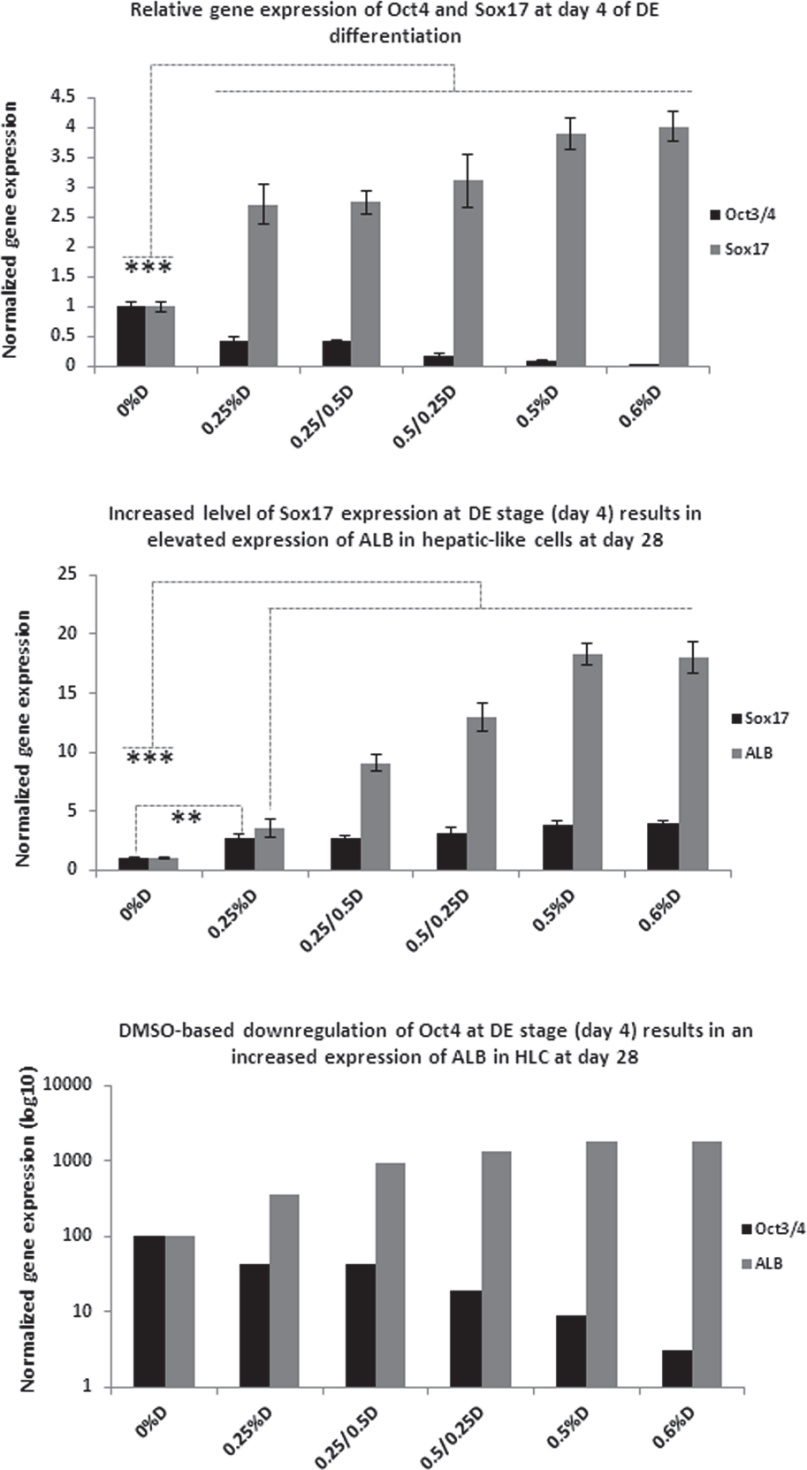
Transcriptional analysis of regulation of OCT4, SOX17 and ALB expression by addition of different concentrations of DMSO to DE medium. (A). Addition of DMSO to the Activin A-based medium during DE formation dose-dependently regulated the correlation between gene expression of the pluripotency marker OCT4 and the DE marker SOX17 during definitive endoderm formation. (B) Expression of the hepatic marker ALB at the end of differentiation linked to the prior expression of SOX17 at day 4 of DE formation. (C) Correlation was also observed between the down regulation of the pluripotency marker OCT4 at day 4 of DE formation and an increase in the expression of ALB at the end of hepatic differentiation. The housekeeping gene GAPDH was used for the initial normalization of the qRT-PCR results. Further standardization to the Activin A culture condition was performed to show relations between all of the investigated conditions. qRT-PCR data is mean ± SD of 3 replicates, Student’s t test: n = 3, (**) p ≤ 0.01, (***) p ≤ 0.001.

To further investigate the optimal concentration of DMSO at specific time points during Activin A-based DE formation and to assess its potency to increase the overall efficiency of hepatocyte differentiation, DE cells were primed through intermediate hepatoblasts to hepatic-like cells. Hepatic-like cells derived from all DE-culture conditions were harvested after 28 days and analysed for expression of the hepatocyte marker albumin (ALB). At day 4 of DE differentiation a dose-dependent correlation was observed between DMSO-induced up regulation of SOX17 and down regulation of OCT4 (Fig. 6A) confirming the beneficial effects of DMSO in the Activin A-driven process of definitive endoderm formation. Moreover, in cultures where 0.5% of DMSO was present during the first 2 days during DE formation followed by 0.25% DMSO during the remaining 2 days, cells showed lower expression of OCT4 than in cultures where the concentrations of DMSO were transposed (0.25% then 0.5%D) or maintained continuously at 0.25%. Down regulated expression of OCT4 in the presence of 0.5% then 0.25% DMSO was paralleled by up regulation of the DE marker SOX17, suggesting that a rapid and efficient decrease of the pluripotency status of cells at the very beginning of commitment to differentiation potentiates their ability to respond to DE-priming factors.

Following the differentiation of hESC to DE, cells were primed further to acquire the identity of hepatic-like cells where analysis of the hepatocyte marker albumin (ALB) at day 28 revealed further advantageous effects of the addition of DMSO to DE-priming medium. Concurrent down regulation of the pluripotency marker OCT4 and up regulation of the DE marker SOX17 after 4 days of DE formation showed strong correlation with the subsequent efficiency of hepatocyte differentiation as evidenced by the increased expression of ALB (Fig. 6B & C).

This observation is of significant importance as it indicates that rapid and complete abolishment of pluripotency via the use of DMSO to modulate chromatin condensation and potentiate expression of key genes during the first days of cellular differentiation has a strong impact on overall efficiency of the specification and differentiation process. These findings may not be linked exclusively to hepatocyte differentiation and may be relevant to differentiation of other DE derived phenotypes, or indeed to specification and differentiation of ectoderm and mesoderm in pursuit of a wide range of differentiated cells from pluripotent sources.

## Discussion

We have developed a simple and robust method for rapid enhancement of the process of DE formation by supplementing Activin A-based culture condition with DMSO. At concentrations of 0.5–0.6% DMSO very rapidly down regulated the otherwise persistent expression of the pluripotency genes OCT4 and NANOG thereby making cells more susceptible to differentiating signals (Fig. 2B, Fig. 6). In parallel with the decrease in the expression of these transcription factors, addition of DMSO to the Activin A-based medium simultaneously increased the expression levels of the DE markers SOX17, GATA4 and CXCR4 (Fig. 2B).

Among the wide range of published methods used to increase the efficiency of definitive endoderm formation, some authors have tried to solve the problem of effectively and rapidly repressing the pluripotency status of cells at the initiation of differentiation. Issues associated with sustained expression of pluripotency genes during initial stages of cellular specification were addressed in the 2010 study by Touboul et al. [13] where H9 human ES cells were differentiated to DE in Activin A-based medium supplemented with the PI3K inhibitor LY294002 and the growth factors FGF2 and BMP4. LY294002 was reported to enhance Activin A signalling; however its effect on the down regulation of the pluripotency status of differentiating cells was not efficient. Touboul et al. reported that only the combination of Activin A with LY294002 (1μM) and the growth factors BMP4 (10ng/ml) and FGF2 (20ng/ml) repressed pluripotency after DE specification was initiated. In the 2012 study by Yu et al. [26] DE specification was achieved by eight days exposure of pluripotent stem cells to 100ng/ml Activin A combined with 20ng/ml FGF2 with 10ng/ml BMP4, however, clusters of OCT4-positive cells were still present, confirming the difficulty of down regulating this pluripotency transcription factor.

Despite the inclusion of BMP and FGF during DE specification in numerous studies, there is a strong risk that activation of signalling pathways by these growth factors at the same time as the NODAL signalling pathway may interfere with the latter and potentiate generation of a mixed or non-specific response in cells. In the 2013 study by Hannan et al. [10], Activin A-based medium was supplemented with FGF2, BMP4 and LY294002 during DE specification, using an experimental design similar to some of the previous studies. However, the concentrations of FGF2 and LY294002 were increased to 100ng/ml and 10μM, respectively, while BMP4 was maintained at 10ng/ml. Use of 100ng/ml FGF2 is confounding as high concentrations of this growth factor are found in a number of pluripotency maintaining media exemplified by the widely used mTeSR. In the 2012 study by Bukong et al. [27] 50ng/ml of FGF2 was added to the Activin A-based DE medium, while FGF4 (20ng/ml), BMP2 (10ng/ml) and LY294002 (25ng/ml) were used in the subsequent hepatoblast priming medium.

Taken together with other previous studies these experimental conditions indicate a focus on a rather limited variety of differentiating factors employed in protocols addressing DE specification. Moreover, some of the difficulties associated with the inability to down regulate pluripotency signalling are likely to arise from the fact that the Activin A-induced NODAL/TGFβ signalling pathway is reported to be involved in both the maintenance of pluripotency and the differentiation of cells towards the endoderm and mesoderm germ layers [28]. In cultures of pluripotent stem cells low concentrations of Activin A (around 10ng/ml) and high concentrations of FGF2 (80–100ng/ml) are routinely present to repress the ability of cells to differentiate, however on initiation of endoderm differentiation, the concentration of Activin A is increased to 100ng/ml. The duality in the role of Activin A may be partially explained by differential regulatory cross-talk between TGFβ signalling and the other, more differentiation stage-specific cascades of signal transduction such as the Wnt signalling pathway [28].

In this study, the addition of 0.5–0.6% of DMSO to Activin A-based medium during DE derivation resulted in a rapid down regulation of the pluripotency genes OCT4 and NANOG (Fig. 2) and as a consequence of this effect DMSO significantly potentiated the ability of Activin A to orchestrate definitive endoderm formation. Use of the HDAC inhibitor sodium butyrate during DE specification has previously been reported by Hay et al. [2], however parallel implementation of our optimised protocol with the Hay et al protocol on the H1 cell line showed improved results with DMSO for effectively down regulating the pluripotency transcription factors OCT4 and NANOG and up regulating SOX17, GATA4 and HHEX (S5 Fig, S6 Fig).

Furthermore, a prominent and surprising feature of using this small molecule during DE specification was the significant enhancement observed in subsequent downstream stages of hepatic differentiation, as shown by the significantly up regulated levels of albumin, suggesting that rapid down regulation of pluripotency genes immediately after initiating cellular differentiation is crucially enabling for cells to efficiently respond to specification signals throughout differentiation. We hypothesize that the direct linear relationship observed between OCT4 down regulation and up regulation of SOX17 at early specification (Day 4, Fig. 6A) results from potentiation of Activin A by DMSO, i.e. rapid and concomitant action on both markers. Early modulation of OCT4 and SOX17 translates at a later time (Day 28, Fig. 6) to an asymptotic relationships between Albumin expression and both markers, where the simple linear relationship observed early in differentiation is modified and constrained by the capacity of the cells for Albumin expression; i.e. a small change in OCT4/SOX17 produces a large change in Albumin 24 days later, with further changes in OCT4/SOX17 having lesser effects. Further evaluation of the mechanism of action of DMSO during DE formation is required to shed more light on its new interesting capability; however, if the histone deacetylase inhibitor property of this small molecule in differentiation DE layer is confirmed, it may also act by enabling certain hepatic transcription factors to access compacted fragments of promoter regions of albumin gene faster than when compared with culture condition where cells are exposed to Activin A only [29]. Additionally, beneficial effects of DMSO on AFP expression during hepatoblast specification were previously reported by Hay et al [2] and (S7 Fig).

Interestingly, Chetty et al [30] demonstrated that a number of hESCs and iPSCs exposed to 1–2% DMSO before initiation of the derivation process showed improved capacity to differentiate into ecto-, endo- and mesoderm. DMSO treatment was shown to increase activation of the retinoblastoma protein and the percentage of cells in the early G1 phase of the cell cycle and may therefore restore checkpoint control of this aspect of the cell cycle in preparation for proliferation and transition to differentiated phenotypes. This pre-treatment approach differs from that of the study reported here, where DMSO treatment at lower concentrations was used in concert with growth directed differentiation and Chetty et al. did not examine the impact of DMSO pre-treatment on the expression of pluripotency genes. At this time it is not known if the actions of DMSO reported by Chetty et al. and those reported here represent separate mechanisms or different aspects of a common mechanism. DMSO may act independently to reset cell cycle control prior to differentiation and to promote down regulation of pluripotency genes after differentiation has been initiated. Alternatively the actions of DMSO observed in the two studies may represent a continuum of actions which prime stem cells for more efficient differentiation. A study examining the combinatorial effects of the timing of DMSO treatment on the expression of pluripotency and differentiation markers would be informative in elucidating this dichotomy.

DMSO-controlled differentiation of definitive endoderm and hepatocyte-like cells therefore provides a novel method for the optimisation of cellular specification and an interesting new insight into the complex interplay between controlling the maintenance of pluripotency and Activin A-driven cellular differentiation. These initial studies indicate that the inability to rapidly down regulate pluripotency genes at the initiation of cellular specification prevents cells from efficiently responding to the differentiating signals and that this key hurdle may be overcome by appropriate potentiation of gene expression with DMSO. Our findings provide a novel simple approach to robust formation of definitive endoderm from pluripotent stem cells by rapidly down regulating the expression levels of pluripotency genes and thereby enhancing the efficacy of downstream cellular differentiations to definitive endoderm derivatives. These findings may have applications beyond hepatocyte differentiation and may provide a new avenue of exploration towards in vivo specification and differentiation of all germ layers to generate a wide range of differentiated cells from pluripotent sources for research and therapeutic use.

## Supporting Information

S1 Fig Chromosomal integrity of human ES cells. Karyotyping of 22 metaphase divisions of human ES cell lines was performed on a regular basis and has showed no abnormality. Here, in H1 human ES cell line male karyotype 46, XY was confirmed by Medical Genetics in University Hospital of Wales, Cardiff.
(PDF)

S2 Fig Pluripotency analysis of undifferentiated human embryonic stem cells. Pluripotency status of human ES cells was routinely investigated in order to ensure optimal quality of starting material. Bright field microscopy (A, B) and immunocytochemistry for Oct4 (C, panels D and E) and SSEA4 (panel E) show undifferentiated integrity of ES cells. DAPI was used to detect nuclei. (A) scale bar 20 μm, (B, C) magnification 4x, (panels D) 200 μm, (panel E) 100 μm.
(PDF)

S3 Fig Embryoid body formation of human ES cells. Expression levels of stage-specific genes were evaluated for embryonic bodies at days 7 and 16 and compared against pluripotent stem cells and hESCs-derived definitive endoderm. (A) Hierarchal clustering performed on heatmap representation of gene expression data revealed that EBs from both of the time-points share the highest similarity in pattern of gene expression. hESCs/human ES cells; DE Day4/definitive endoderm differentiated via optimised protocol; EB day7 and EB day16/embryonic bodies harvested at days 7 and 16, respectively. Bar chart analysis of levels of endoderm genes (B) and the pluripotency markers (C) illustrate strong commitment of EB to the differentiation process. (D) Embryoid bodies derived from human ES cell line were robustly generated only if ROCK inhibitor was added during the initial stage of EBs formation. Scale bar, 200μm.
(PDF)

S4 Fig Immunofluorescence staining of OCT4 and SOX17 at day 4 of DE specification. Addition of 30μM of the TGFb signalling inhibitor SB-431542 to the Activin A-driven differentiation abolished the ability of Activin A to induce cells to express SOX17. ‘Medium only’ represents culture condition deprived of differentiating signals to monitor spontaneous differentiation. Activin A and ActivinA+0.5%DMSO were used as positive controls for DE specification. DAPI was used to stain nuclei. Scale bar 100μm.
(PDF)

S5 Fig Transcriptional analysis of definitive endoderm cells derived from hESC using the optimised DMSO protocol (KCGE) and the Hay et al. [2] (Hay) protocols. (A) Cells were analysed for the expression of the pluripotency markers OCT4 and NANOG and the definitive endoderm markers, SOX17, HHEX, GSC, GATA4, FOXA2 and CXCR4. Expression levels of the mesendoderm/early mesoderm marker Brachyury (T) and extra-embryonic SOX7 and AFP markers were also monitored. Euclidean-based clustering grouped DE cells derived by the KCGE protocol separately from DE cells differentiated via the Hay protocol. The HepG2 cell line was used as a partial negative control for differentiation, and showed the expected expression of AFP, FOXA2 and HHEX and absence of pluripotency and DE markers. (B) Applying the KCGE protocol for DE formation resulted in cells expressing significantly higher levels of stage-specific transcription factors analysed via qRT-PCR than when cells were differentiated using the Hay et al. protocol. Student’s t test: n = 3, (***) p ≤ 0.001, (**) p ≤ 0.01
(PDF)

S6 Fig Immunofluorescence staining of DE for OCT4 and SOX17. hESC were differentiated to DE via the KCGE protocol (A) and the Hay et al. protocol (2008). (B). Scale bar 100 μm.
(PDF)

S7 Fig Addition of DMSO to hepatoblast cocktail of growth factors can increase level of AFP expression. HESCs-derived and ActivinA/0.5%DMSO-treated definitive endoderm cells were primed for subsequent eight days to the stage of hepatoblasts in a variety of culture media. AFP expression in control culture condition based on the Hay et al. protocol [2] for hepatoblast formation is shown as 1% DMSO. B/BMP2 (30ng/ml); F/FGF4 (10ng/ml); H/HGF (10ng/ml), D/DMSO (0.5%). The housekeeping gene GAPDH was used for normalization of raw qRT-PCR results. Student’s t test: n = 3, (**) *p* ≤ 0.01, (***) *p* ≤ 0.001
(PDF)
