# Supplementary material for: DMSO Efficiently Down Regulates Pluripotency Genes in Human Embryonic Stem Cells during Definitive Endoderm Derivation and Increases the Proficiency of Hepatic Differentiation
Source: PLoS One. 2015 Feb 6;10(2):e0117689. doi: 10.1371/journal.pone.0117689 (PMC4320104; doi:10.1371/journal.pone.0117689)
Supplement: S1 Fig — Karyotyping of 22 metaphase divisions of human ES cell lines was performed on a regular basis and has showed no abnormality. Here, in H1 human ES cell line male karyotype 46, XY was confirmed by Medical Genetics in University Hospital of Wales, Cardiff. (PDF) [file pone.0117689.s001.pdf]

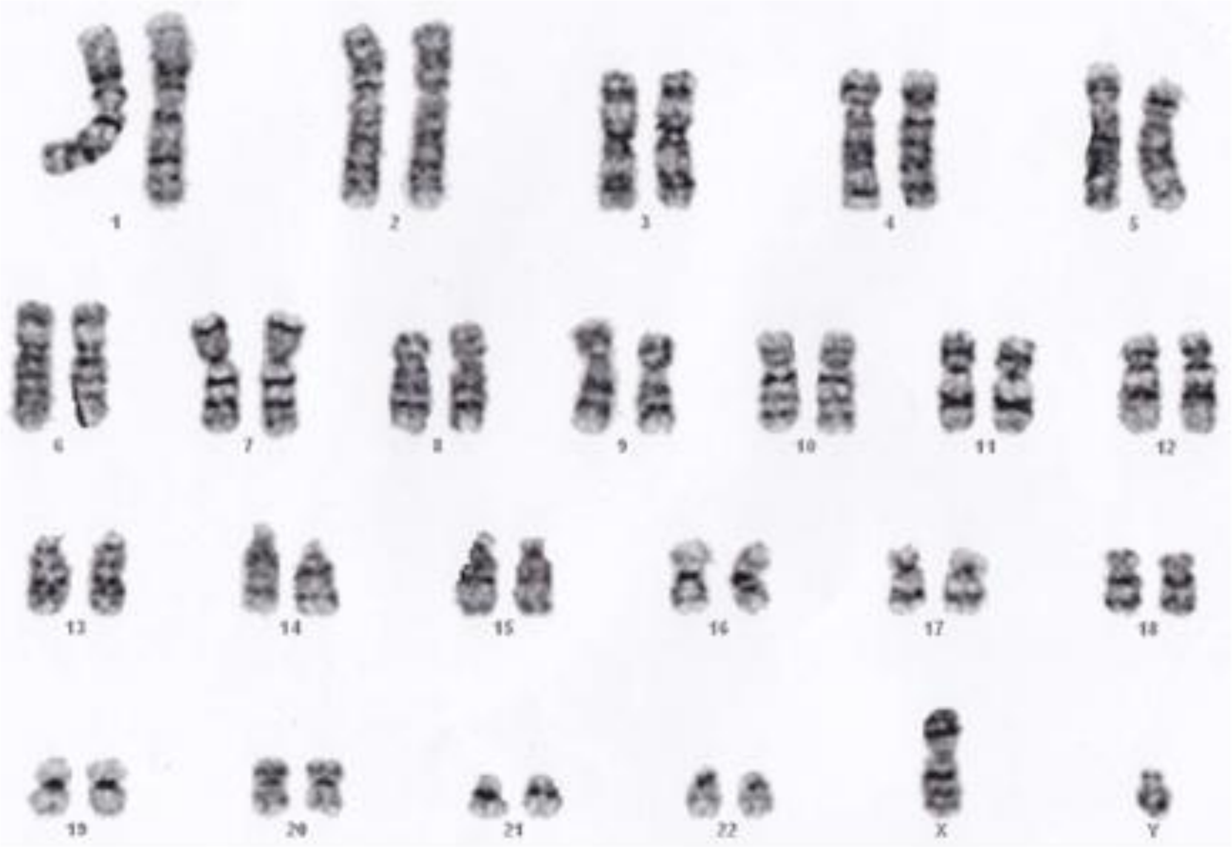

S1 Figure: Chromosomal integrity of human ES cells.

Karyotyping of 22 metaphase divisions of human ES cell lines was performed on a regular basis and has showed no abnormality. Here, in H1 human ES cell line male karyotype 46, XY was confirmed by Medical Genetics in University Hospital of Wales, Cardiff.
