# Supplementary material for: DMSO Efficiently Down Regulates Pluripotency Genes in Human Embryonic Stem Cells during Definitive Endoderm Derivation and Increases the Proficiency of Hepatic Differentiation
Source: PLoS One. 2015 Feb 6;10(2):e0117689. doi: 10.1371/journal.pone.0117689 (PMC4320104; doi:10.1371/journal.pone.0117689)
Supplement: S2 Fig — Pluripotency status of human ES cells was routinely investigated in order to ensure optimal quality of starting material. Bright field microscopy (A, B) and immunocytochemistry for Oct4 (C, panels D and E) and SSEA4 (panel E) show undifferentiated integrity of ES cells. DAPI was used to detect nuclei. (A) scale bar 20 μm, (B, C) magnification 4x, (panels D) 200 μm, (panel E) 100 μm. (PDF) [file pone.0117689.s002.pdf]

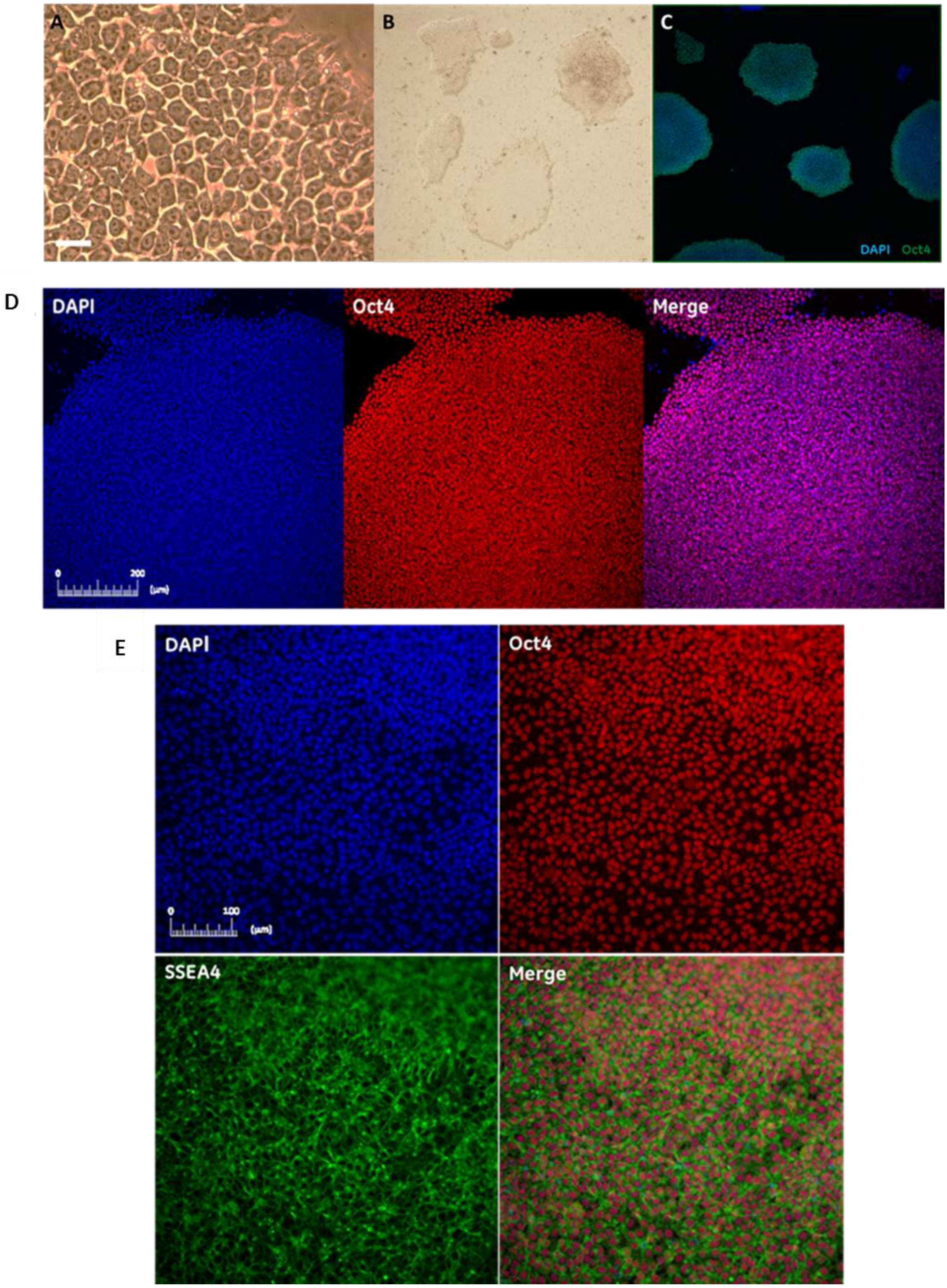


**S2 Figure: Pluripotency analysis of undifferentiated human embryonic stem cells.**

Pluripotency status of human ES cells was routinely investigated in order to ensure optimal quality of starting material. Bright field microscopy (A, B) and immunocytochemistry for Oct4 (C, panels D and E) and SSEA4 (panel E) show undifferentiated integrity of ES cells. DAPI was used to detect nuclei. (A) scale bar 20 µm, (B, C) magnification 4x, (panels D) 200 µm, (panel E) 100 µm.
