# Supplementary material for: DMSO Efficiently Down Regulates Pluripotency Genes in Human Embryonic Stem Cells during Definitive Endoderm Derivation and Increases the Proficiency of Hepatic Differentiation
Source: PLoS One. 2015 Feb 6;10(2):e0117689. doi: 10.1371/journal.pone.0117689 (PMC4320104; doi:10.1371/journal.pone.0117689)
Supplement: S3 Fig — Expression levels of stage-specific genes were evaluated for embryonic bodies at days 7 and 16 and compared against pluripotent stem cells and hESCs-derived definitive endoderm. (A) Hierarchal clustering performed on heatmap representation of gene expression data revealed that EBs from both of the time-points share the highest similarity in pattern of gene expression. hESCs/human ES cells; DE Day4/definitive endoderm differentiated via optimised protocol; EB day7 and EB day16/embryonic bodies harvested at days 7 and 16, respectively. Bar chart analysis of levels of endoderm genes (B) and the pluripotency markers (C) illustrate strong commitment of EB to the differentiation process. (D) Embryoid bodies derived from human ES cell line were robustly generated only if ROCK inhibitor was added during the initial stage of EBs formation. Scale bar, 200μm. (PDF) [file pone.0117689.s003.pdf]

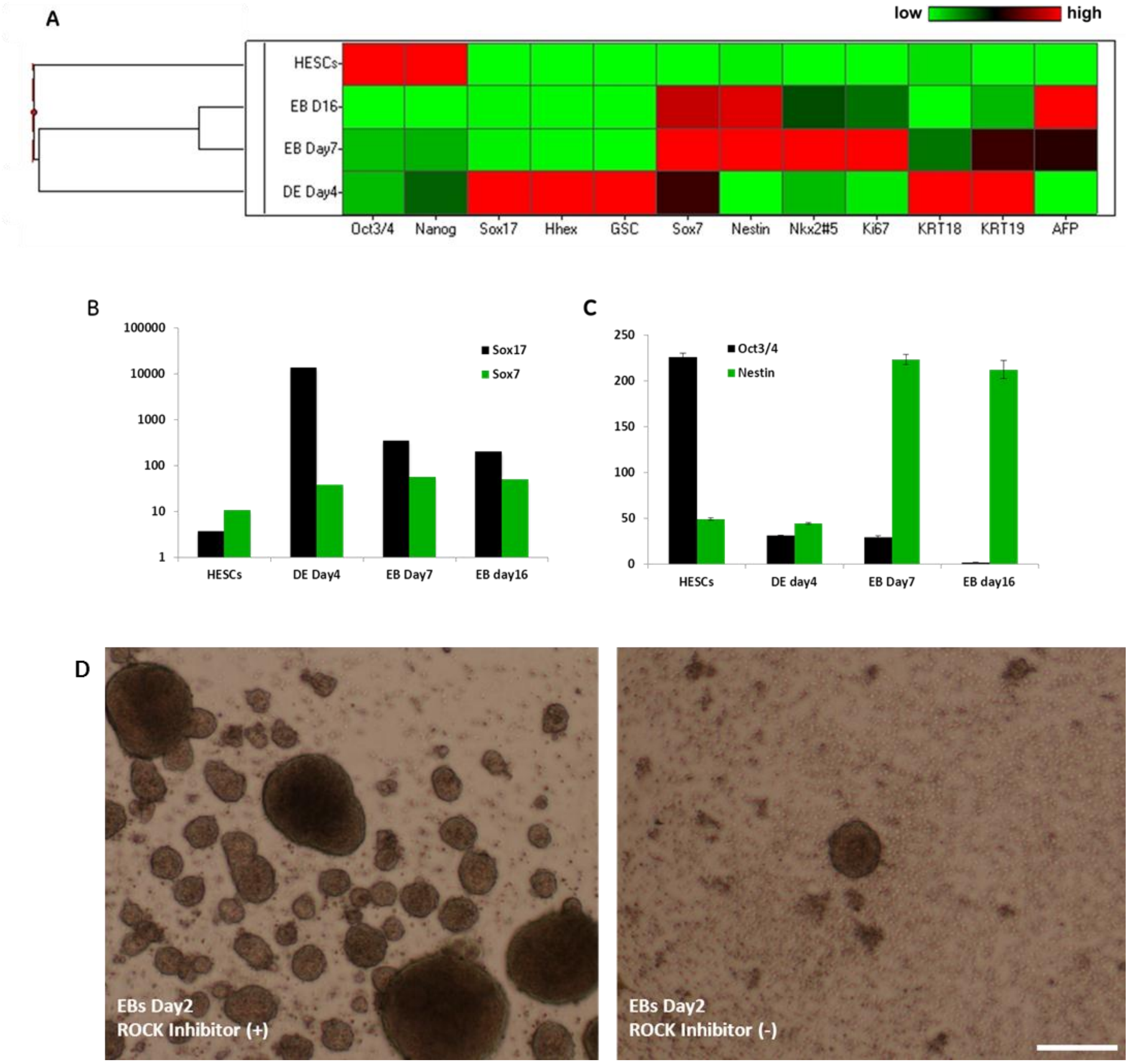

S3 Figure: Embryoid body formation of human ES cells.

Expression levels of stage-specific genes were evaluated for embryonic bodies at days 7 and 16 and compared against pluripotent stem cells and hESCs-derived definitive endoderm. (A) Hierarchal clustering performed on heatmap representation of gene expression data revealed that EBs from both of the time-points share the highest similarity in pattern of gene expression. hESCs/human ES cells; DE Day4/definitive endoderm differentiated via optimised protocol; EB day7 and EB day16/embryonic bodies harvested at days 7 and 16, respectively. Bar chart analysis of levels of endoderm genes (B) and the pluripotency markers (C) illustrate strong commitment of EB to the differentiation process. (D) Embryoid bodies derived from human ES cell line were robustly generated only if ROCK inhibitor was added during the initial stage of EBs formation. Scale bar, 200µm.
