# Supplementary material for: DMSO Efficiently Down Regulates Pluripotency Genes in Human Embryonic Stem Cells during Definitive Endoderm Derivation and Increases the Proficiency of Hepatic Differentiation
Source: PLoS One. 2015 Feb 6;10(2):e0117689. doi: 10.1371/journal.pone.0117689 (PMC4320104; doi:10.1371/journal.pone.0117689)
Supplement: S4 Fig — Addition of 30μM of the TGFb signalling inhibitor SB-431542 to the Activin A-driven differentiation abolished the ability of Activin A to induce cells to express SOX17. ‘Medium only’ represents culture condition deprived of differentiating signals to monitor spontaneous differentiation. Activin A and ActivinA+0.5%DMSO were used as positive controls for DE specification. DAPI was used to stain nuclei. Scale bar 100μm. (PDF) [file pone.0117689.s004.pdf]

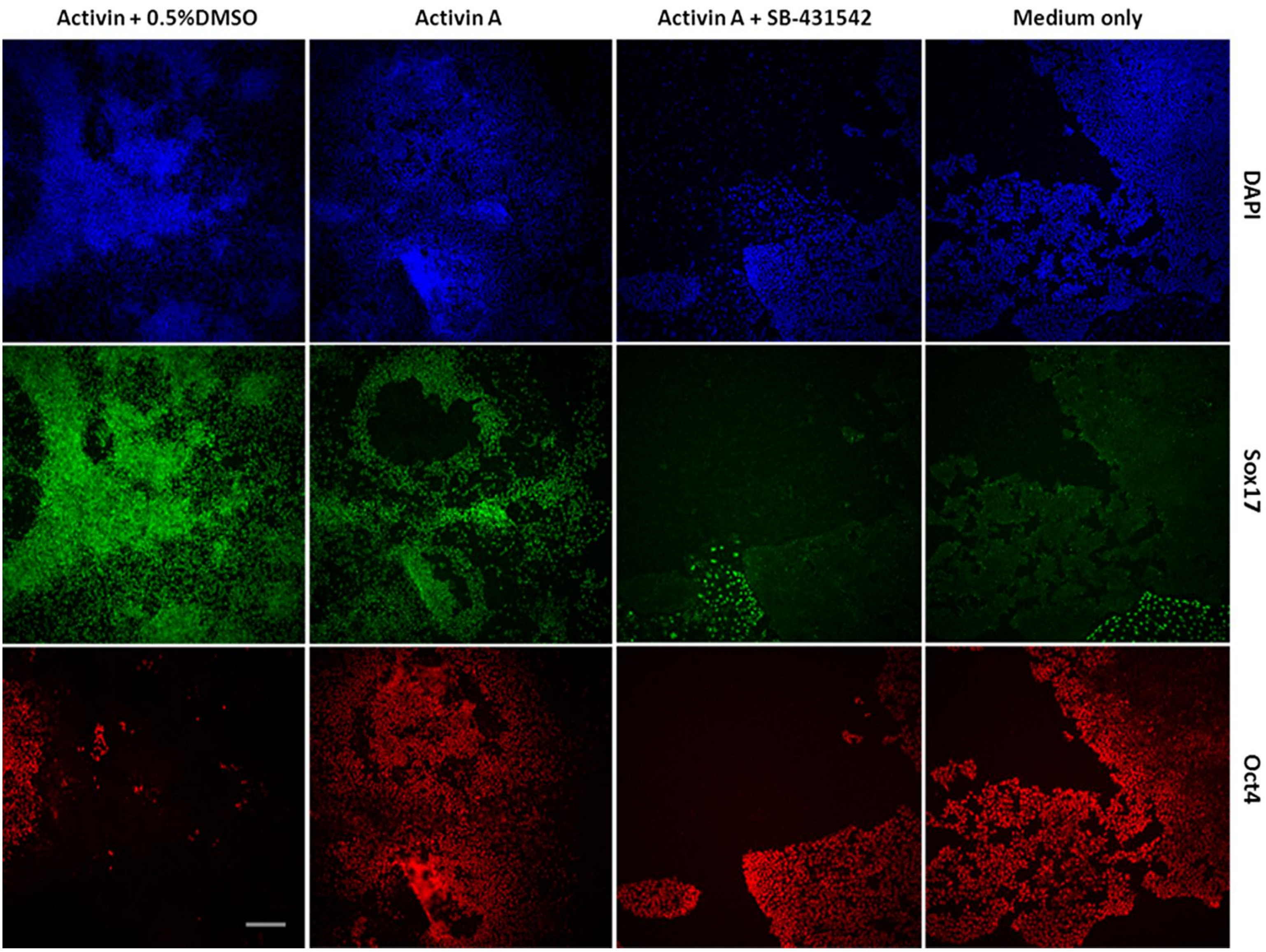

S4 Figure: Immunofluorescence staining of OCT4 and OX17 at day 4 of DE specification.

Addition of 30µM of the TGFb signalling inhibitor SB-431542 to the Activin A-driven differentiation abolished the ability of Activin A to induce cells to express SOX17. 'Medium only' represents culture condition deprived of differentiating signals to monitor spontaneous differentiation. Activin A and ActivinA+0.5%DMSO were used as positive controls for DE specification. DAPI was used to stain nuclei. Scale bar 100µm.
