# Supplementary material for: DMSO Efficiently Down Regulates Pluripotency Genes in Human Embryonic Stem Cells during Definitive Endoderm Derivation and Increases the Proficiency of Hepatic Differentiation
Source: PLoS One. 2015 Feb 6;10(2):e0117689. doi: 10.1371/journal.pone.0117689 (PMC4320104; doi:10.1371/journal.pone.0117689)
Supplement: S5 Fig — (A) Cells were analysed for the expression of the pluripotency markers OCT4 and NANOG and the definitive endoderm markers, SOX17, HHEX, GSC, GATA4, FOXA2 and CXCR4. Expression levels of the mesendoderm/early mesoderm marker Brachyury (T) and extra-embryonic SOX7 and AFP markers were also monitored. Euclidean-based clustering grouped DE cells derived by the KCGE protocol separately from DE cells differentiated via the Hay protocol. The HepG2 cell line was used as a partial negative control for differentiation, and showed the expected expression of AFP, FOXA2 and HHEX and absence of pluripotency and DE markers. (B) Applying the KCGE protocol for DE formation resulted in cells expressing significantly higher levels of stage-specific transcription factors analysed via qRT-PCR than when cells were differentiated using the Hay et al. protocol. Student’s t test: n = 3, (***) p ≤ 0.001, (**) p ≤ 0.01 (PDF) [file pone.0117689.s005.pdf]

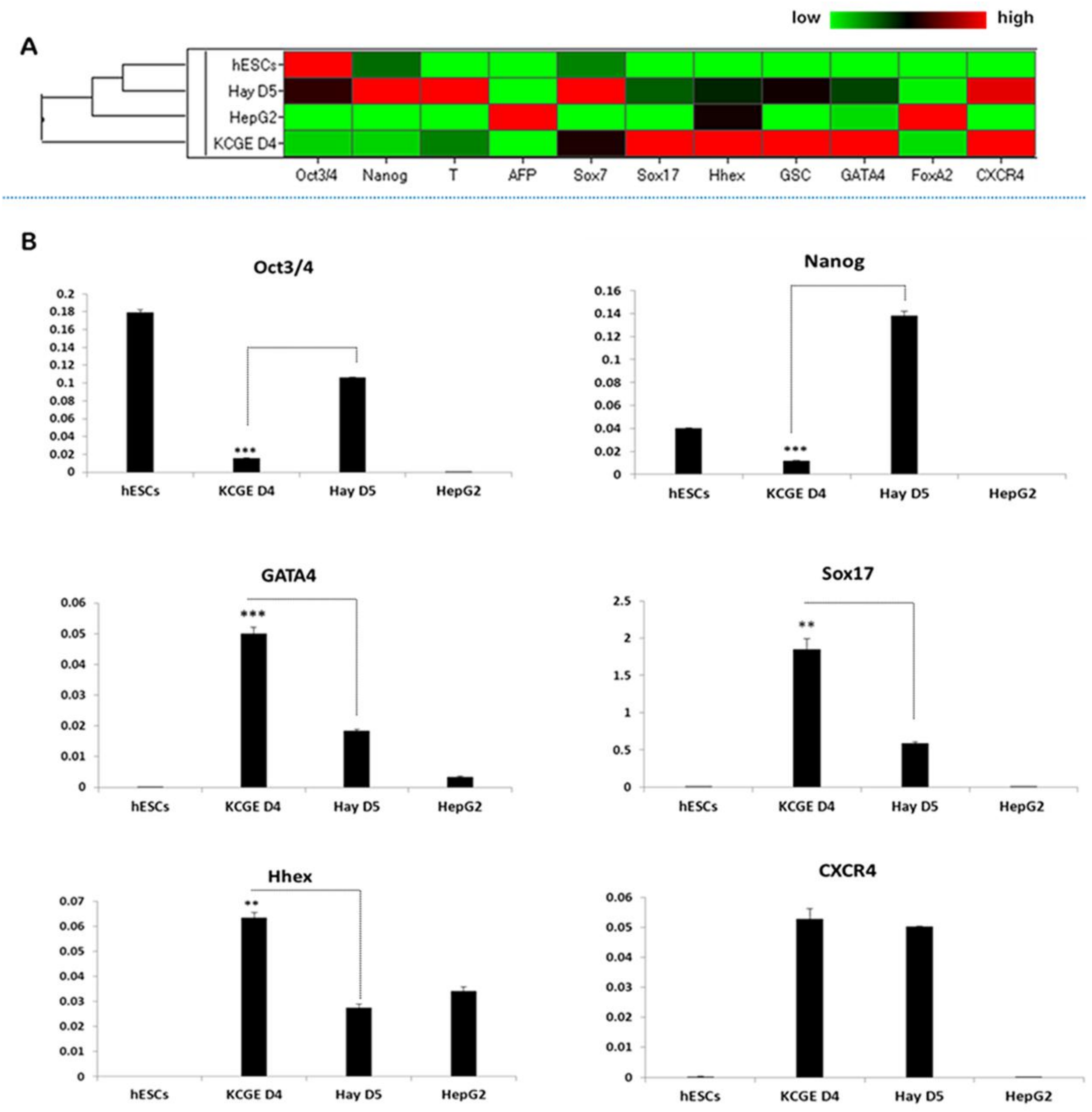

S5 Figure: Transcriptional analysis of definitive endoderm cells derived from hESC using the optimised DMSO protocol (KCGE) and the Hay et al. [2] (Hay) protocols.

(A) Cells were analysed for the expression of the pluripotency markers OCT4 and NANOG and the definitive endoderm markers, SOX17, HHEX, GSC, GATA4, FOXA2 and CXCR4. Expression levels of the mesendoderm/early mesoderm marker Brachyury (T) and extra-embryonic SOX7 and AFP markers were also monitored. Euclidean-based clustering grouped DE cells derived by the KCGE protocol separately from DE cells differentiated via the Hay protocol. The HepG2 cell line was used as a partial negative control for differentiation, and showed the expected expression of AFP, FOXA2 and HHEX and absence of pluripotency and DE markers. (B) Applying the KCGE protocol for DE formation resulted in cells expressing significantly higher levels of stage-specific transcription factors analysed via qRT-PCR than when cells were differentiated using the Hay et al. protocol. Student's t test: n=3, (***) $p \leq 0.001$, (**) $p \leq 0.01$
