# Supplementary material for: DMSO Efficiently Down Regulates Pluripotency Genes in Human Embryonic Stem Cells during Definitive Endoderm Derivation and Increases the Proficiency of Hepatic Differentiation
Source: PLoS One. 2015 Feb 6;10(2):e0117689. doi: 10.1371/journal.pone.0117689 (PMC4320104; doi:10.1371/journal.pone.0117689)
Supplement: S6 Fig — hESC were differentiated to DE via the KCGE protocol (A) and the Hay et al. protocol (2008). (B). Scale bar 100 μm. (PDF) [file pone.0117689.s006.pdf]

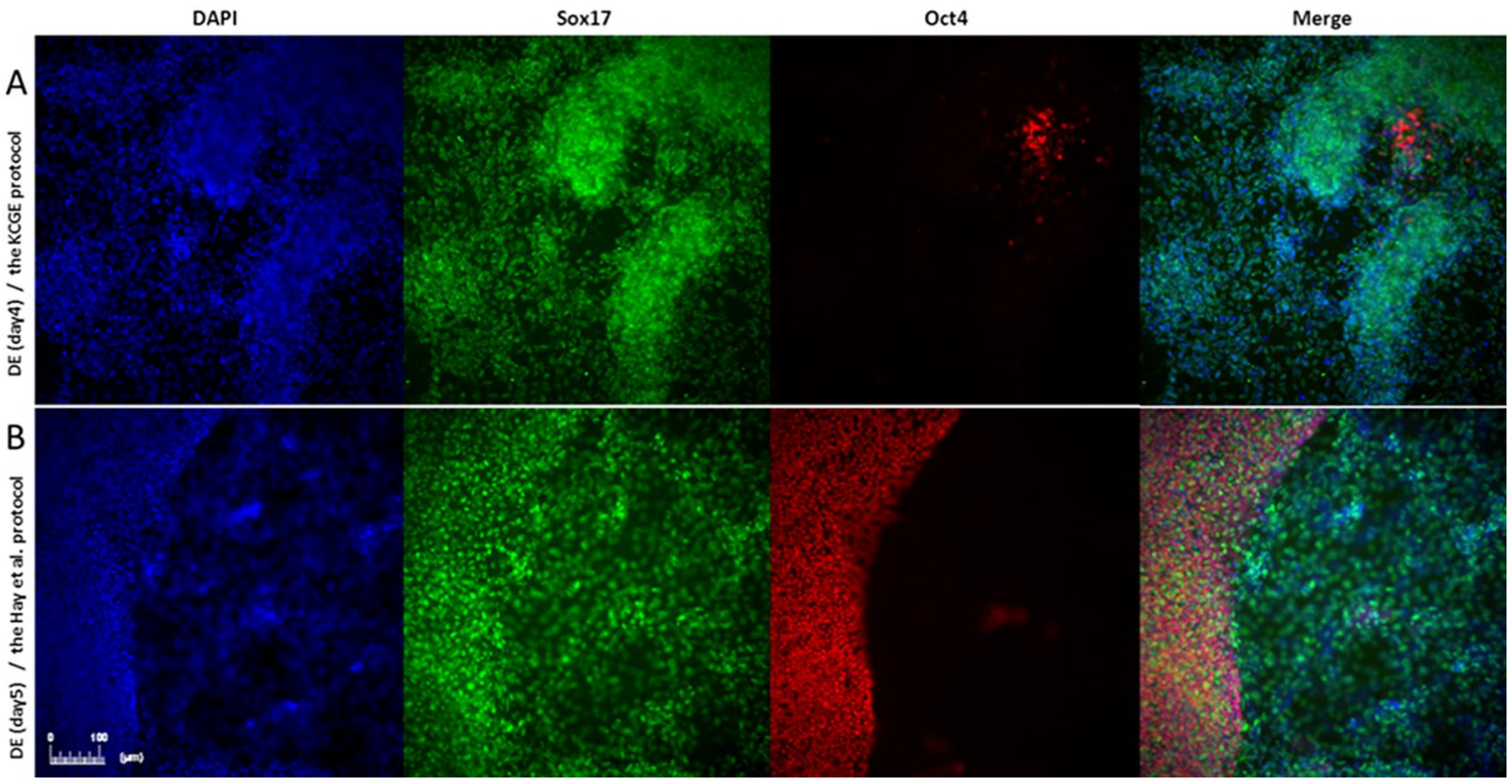

S6 Figure: Immunofluorescence staining of DE for OCT4 and SOX17.

hESC were differentiated to DE via the KCGE protocol (A) and the Hay et al. protocol (2008). (B). Scale bar 100 µm.
