# Supplementary material for: DMSO Efficiently Down Regulates Pluripotency Genes in Human Embryonic Stem Cells during Definitive Endoderm Derivation and Increases the Proficiency of Hepatic Differentiation
Source: PLoS One. 2015 Feb 6;10(2):e0117689. doi: 10.1371/journal.pone.0117689 (PMC4320104; doi:10.1371/journal.pone.0117689)
Supplement: S7 Fig — HESCs-derived and ActivinA/0.5%DMSO-treated definitive endoderm cells were primed for subsequent eight days to the stage of hepatoblasts in a variety of culture media. AFP expression in control culture condition based on the Hay et al. protocol [2] for hepatoblast formation is shown as 1% DMSO. B/BMP2 (30ng/ml); F/FGF4 (10ng/ml); H/HGF (10ng/ml), D/DMSO (0.5%). The housekeeping gene GAPDH was used for normalization of raw qRT-PCR results. Student’s t test: n = 3, (**) p ≤ 0.01, (***) p ≤ 0.001 (PDF) [file pone.0117689.s007.pdf]

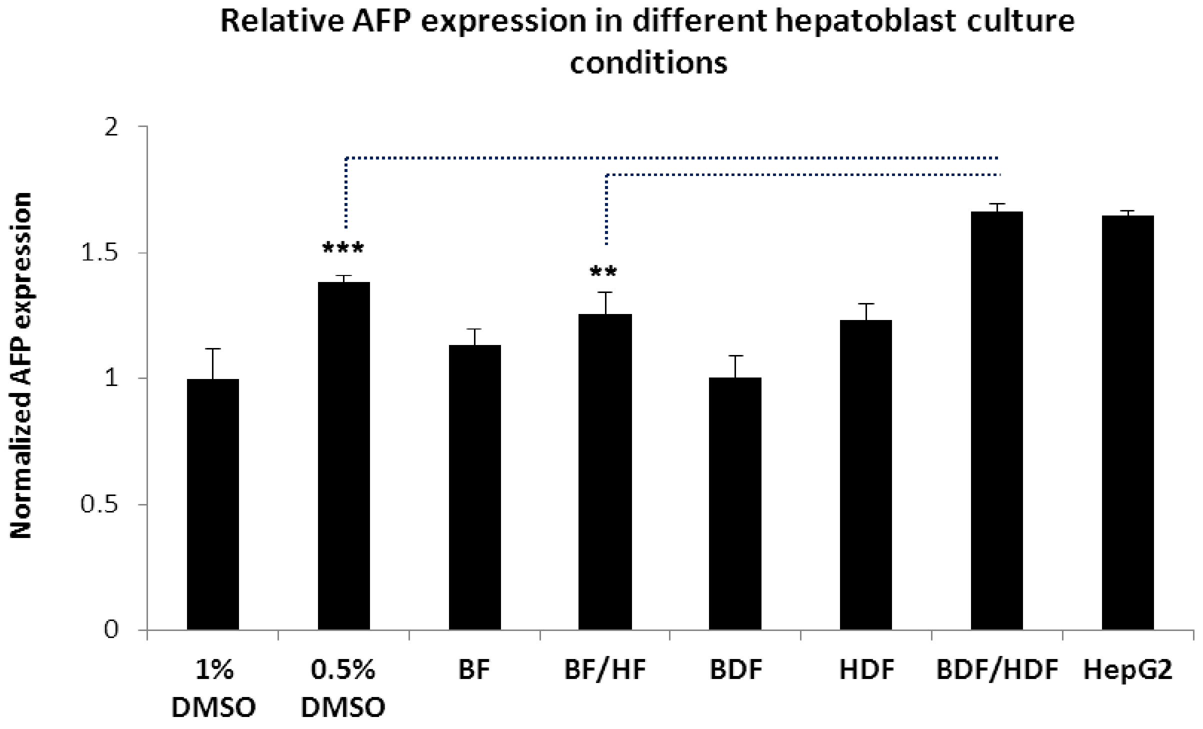

S7 Figure: Addition of DMSO to hepatoblast cocktail of growth factors can increase level of AFP expression.

HESCs-derived and ActivinA/0.5%DMSO-treated definitive endoderm cells were primed for subsequent eight days to the stage of hepatoblasts in a variety of culture media. AFP expression in control culture condition based on the Hay et al. protocol [2] for hepatoblast formation is shown as 1% DMSO. B/BMP2 (30ng/ml); F/FGF4 (10ng/ml); H/HGF (10ng/ml), D/DMSO (0.5%). The housekeeping gene GAPDH was used for normalization of raw qRT-PCR results. Student's t test: n=3, (**) $p \leq 0.01$, (***) $p \leq 0.001$
